# Expression of organic cation transporter 3 (SLC22A3) and plasma membrane monoamine transporter (SLC29A4) in human umbilical vein endothelial cells and their relevance for histamine uptake

**DOI:** 10.1186/2050-6511-13-S1-A73

**Published:** 2012-09-17

**Authors:** Polonca Ferk, Metoda Lipnik-Štangelj, Mojca Kržan, Katarina Černe

**Affiliations:** 1 Department of Pharmacology and Experimental Toxicology, Faculty of Medicine, University of Maribor, 2000 Maribor, Slovenia; 2 Institute of Pharmacology and Experimental Toxicology, Faculty of Medicine, University of Ljubljana, 1000 Ljubljana, Slovenia

## Background

Increased plasma histamine levels lead to pathological events. Endothelial cells actively participate in histamine clearance by promoting its uptake via yet unidentified carriers, thus limiting histamine effects. The organic cation transporter 3 (OCT3) and plasma membrane monoamine transporter (PMAT) are the two most prominent transporters for endogenous monoamines. OCT3 and PMAT show Na^+^/K^+^-ATPase independency. Both are highly sensitive to inhibition by the isocyanine compound, decynium-22. However, OCT3 is highly sensitive to corticosteron, whereas PMAT is not. We showed in the past that decynium-22 inhibits histamine uptake in cultured human umbilical vascular endothelial cells (HUVEC). In the present study we identified the expression of OCT3 and PMAT in freshly isolated and cultured HUVEC as well as some characteristics of histamine uptake in cultured HUVEC such as ouabain and corticosteron sensitivity.

## Methods

We used freshly isolated and cultured HUVEC for determination of mRNA levels of hOCT3 and hPMAT transporters by real-time PCR. For the histamine uptake studies we used cultured HUVEC and determined [^3^H]histamine uptake.

## Results

OCT3 and PMAT are expressed in freshly isolated HUVEC as well as in primary HUVEC culture. Ouabain (0.1 mM) had no influence on uptake of histamine. Corticosteron inhibited the uptake of histamine in HUVEC, however the effect was observed only in mM concentration.

## Conclusions

Our results suggest that because of low sensitivity of histamine uptake to corticosteron, expression of OCT3 in HUVEC is probably not relevant to histamine uptake in these cells, while PMAT expression is worthy of further examination.

